# Molecular Cloning Designer Simulator (MCDS): All-in-one molecular cloning and genetic engineering design, simulation and management software for complex synthetic biology and metabolic engineering projects

**DOI:** 10.1016/j.meteno.2016.05.003

**Published:** 2016-05-12

**Authors:** Zhenyu Shi, Claudia E. Vickers

**Affiliations:** Australian Institute for Bioengineering & Nanotechnology, The University of Queensland, St Lucia, QLD 4072, Australia

**Keywords:** BioCAD, Genetic engineering software, Molecular cloning software, Synthetic biology, Workflow simulation and management

## Abstract

Molecular Cloning Designer Simulator (MCDS) is a powerful new all-in-one cloning and genetic engineering design, simulation and management software platform developed for complex synthetic biology and metabolic engineering projects. In addition to standard functions, it has a number of features that are either unique, or are not found in combination in any one software package: (1) it has a novel interactive flow-chart user interface for complex multi-step processes, allowing an integrated overview of the whole project; (2) it can perform a user-defined workflow of cloning steps in a single execution of the software; (3) it can handle multiple types of genetic recombineering, a technique that is rapidly replacing classical cloning for many applications; (4) it includes experimental information to conveniently guide wet lab work; and (5) it can store results and comments to allow the tracking and management of the whole project in one platform. MCDS is freely available from https://mcds.codeplex.com.

## Highlights

• MCDS is an all-in-one in silico design, simulation and management platform.
• MCDS supports the design, simulation management of most cloning and recombineering technologies.
• MCDS has a novel interactive flowchart that allows simpler and more precise transactions.
• MCDS enables complete information integrity and consistency by keeping all details in one file.

## 1 Background

With the development of synthetic biology and metabolic engineering, DNA manipulations have become increasingly more complex. In addition to classical cloning, workflows may include homologous recombination (Court et al., 2002), lambda Red recombination (Datsenko and Wanner, 2000, Sabri et al., 2013, Stringer et al., 2012), landing-pad integration (Kuhlman and Cox, 2010), site-specific recombination (Haldimann and Wanner, 2001, Shi et al., 2013), ΦC31 assembly (Colloms et al., 2014), CLIVA assembly (Zou et al., 2013), in vivo CRISPR digestion (Jiang et al., 2013), etc. Moreover, synthetic pathway optimization often relies heavily on screening a range of different combinations (Jang et al., 2012, Kirby and Keasling, 2008, Steen et al., 2008), requiring adjustments to the workflow in high-throughput systems.

The technology for designing and managing molecular cloning experiments has undergone significant evolution in the last 30 years. Early design and management was done manually based on sequence text – a painstaking, low-throughput process. In the late 1990s and early 2000s, some software programs for displaying plasmid vector maps and performing individual tasks became available. These included: the early versions of Vector NTI (Lu and Moriyama, 2004), DNAman (lynnon, 1995) or ApE (Davis, 2006), and Clone Manager (Software), which could be used for importing sequences and checking DNA maps; NEBcutter (New England Biolabs) for analyzing restriction sites; Primer Premier (PremierBiosoft) for designing primers; GeneDesign 3.0 (Richardson et al., 2010), Gene Designer (Villalobos et al., 2006), OPTIMIZER (Puigbo et al., 2007), DNAWorks (Hoover and Lubkowski, 2002) or Gene2Oligo (Rouillard et al., 2004) for optimizing gene codons for synthesis; and BioEdit (Hall, 1999 ) for viewing and confirming sequencing data. Cloning design required use of two or more of these tools; copy-paste was used to build new sequences, and word files, lab notes, lists of images and sequencing data files were used to manage and track the experiments.

In recent years, standalone or online software programs that provide better support for the design and construction of commonly used methods became available. These packages can handle one or more techniques, including recombinant DNA technology (Jackson et al., 1972), Gibson Assembly (Gibson, 2011, Gibson et al., 2009), BioBricks construction (Shetty et al., 2008), Golden Gate cloning (Engler et al., 2009, Engler and Marillonnet, 2014, Marillonnet and Werner, 2015), Gateway Assembly (Freuler et al., 2008, Hartley et al., 2000), In-Fusion cloning (Sleight et al., 2010), Sequence and Ligation Independent Cloning (SLIC) (Li and Elledge, 2007), yeast homologous assembly (Noskov et al., 2003) and recET homologous recombination (Fu et al., 2012). Programs include later versions of VectorNTI (Lu and Moriyama, 2004), Clone Manager (Software), J5 (Hillson et al., 2012), DeviceEditor (Chen et al., 2012), VectorEditor (Ham et al., 2012) Benchling (Benchling), GenomeCompiler (Genome Compiler), SnapGene (GSL Biotech), Geneious (Biomatters), etc. Such programs typically include user interfaces for generating enzyme digestion/PCR fragments, which are stored as a list of files. Another user interface is used to select fragment files from the list, assemble the selected fragments into new plasmids, and save the new plasmids in the file list. In some packages, a brief description of the operation procedures is included for each file.

While these programs represent a substantial step forwards over manual curation, the existing programs still suffer from significant drawbacks in the context of managing the complex processes that are now routine in modern molecular biology. We identified the following major limitations: 1) an inability to simulate and overview multiple steps in one project; 2) an inability to introduce corrections/updates early in the design process and have them propagated throughout the project (e. g. if a modification in a primer of the first file is made, the user must manually update all downstream files in the workflow); 3) no/poor support for genomic recombineering technologies such as homologous recombination (Court et al., 2002), Lambda Red recombination (Datsenko and Wanner, 2000, Sabri et al., 2013, Stringer et al., 2012), Landing-Pad integration (Kuhlman and Cox, 2010), site-specific recombination (Haldimann and Wanner, 2001, Shi et al., 2013); and 4) no/poor support for tracking and management of the whole project. To address these limitations, we developed a powerful new all-in-one software platform that allows project level design, simulation, and management, with a novel interactive flowchart user interface. The software, Molecular Cloning and Design Simulator (MCDS; previously called Vexcutor) is now freely available for academics at https://mcds.codeplex.com.

## 2 Materials and methods

Visual Basic (VB) was used as the programming language. The code was written de novo; the current version for download was compiled from 476 source files with 109,558 lines of novel code. The software was originally based on Microsoft. Net Framework 4.0, and was updated to the Microsoft. Net Framework 4.6 to comply with Windows 10 upgrades. WinForm and Windows Presentation Foundation (WPF) were used to develop Graphic User Interface (GUI). The flowchart view, DNA sequence view and vector map view were developed with the WinFrom Graphics Device Interface (GDI). Each step of a whole project is presented as a node in the flowchart view. The vector map is presented if the node contains only one vector. Details of all vector maps can be found in the'detail property' view.

## 3 Features, functions, and instructions

**Fig. 1:**
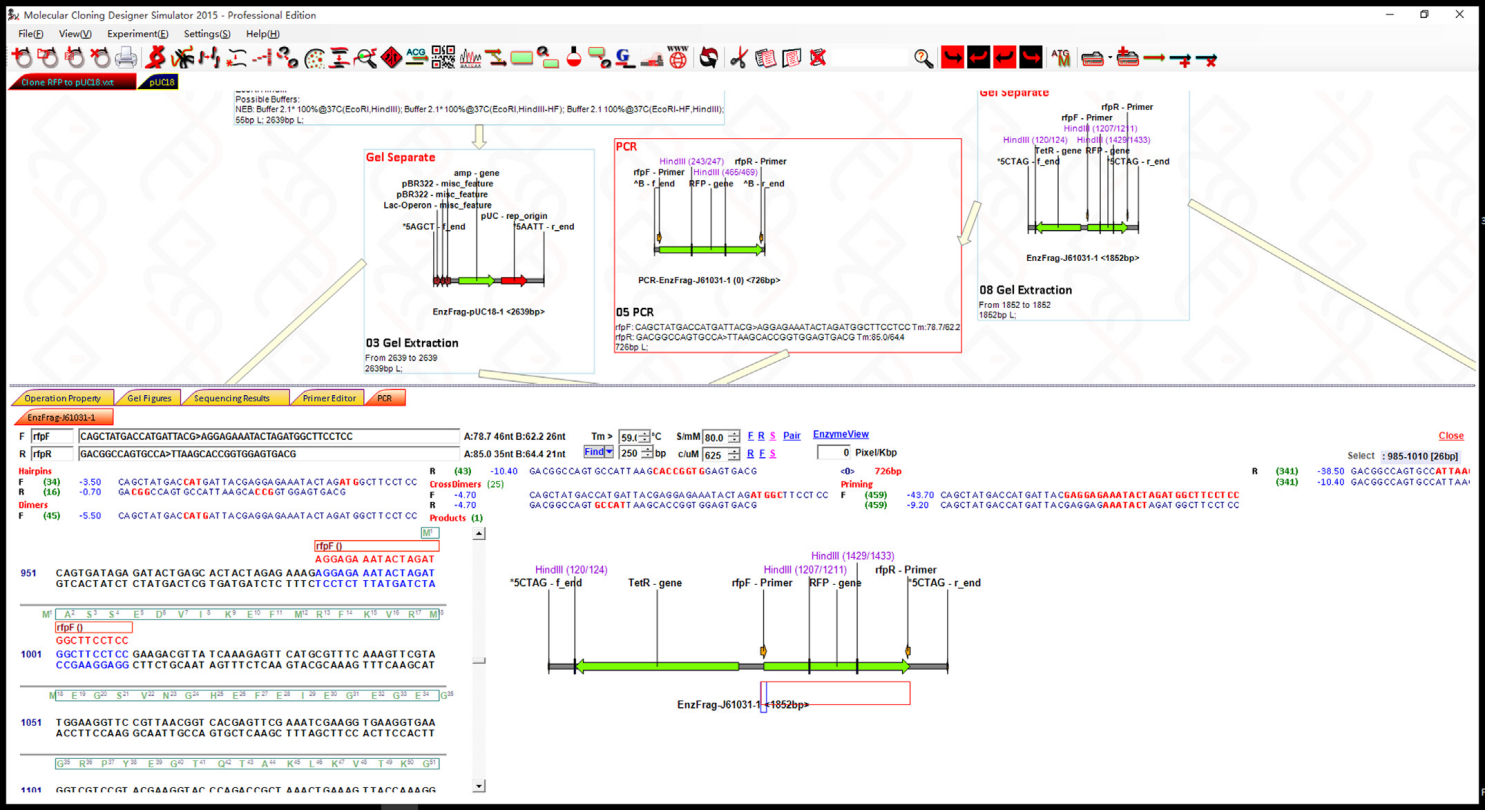
Overview of the MCDS user interface.

**Fig. 2:**
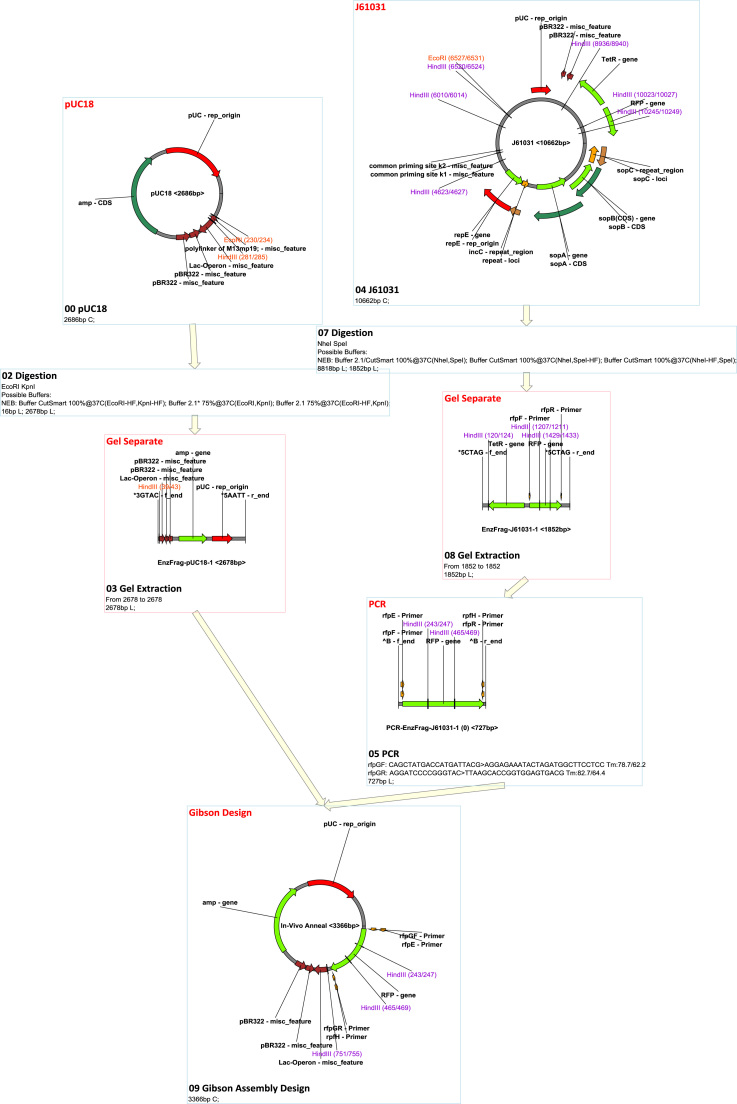
MCDS design and simulation of sample project.

MCDS has a facile graphic user interface (GUI) where functions are provided though both toolbar buttons and in drop-down menus (Fig. 1). The supported functions (‘node’ operations; see below) and other features of the software are outlined in Table 1, Table 2. Features can be accessed from various menus and panels within the GUI (see below for details). MCDS was used in a previous publication to design, simulate and manage complex plasmid construction and lambda red recombination, site-specific recombination (Shi et al., 2013). For the purposes of this description, MCDS was applied to simulate molecular cloning and genetic modification by recombineering (Fig. 2 and Supplementary file 1). It has been used routinely in our lab for day-to-day design, and has been downloaded for use in a number of other laboratories. The software can be downloaded from https://mcds.codeplex.com and tutorial videos can be found both on the start page of the software and online at https://goo.gl/4UUmiH.

**Table 1:** Supported functions (Operations/nodes). These functions are available as buttons on the main menu bar at the top of the GUI.

**Function (Node)**	**Comments**
DNA sequence	Load DNA sequence files into MCDS flowchart to create a new node
Restriction digestion	Use DNA in selected node(s) as substrate(s) for restriction digestion. If sites of two or more enzymes overlap, MCDS will warn user about the confliction
PCR	Use DNA in selected node(s) as template to perform PCR
	MCDS will generate single strand intermediates and then calculating the double strand products by annealing the intermediates. Therefore, MCDS generates by-products to allow users to understand the performance of their primers.
Modification	Apply CIAP (calf intestinal alkaline phosphatase)/Klenow Fragment blunting/T4 DNA polymerase blunting/polynucleotide kinase to selected nodes
Gel electrophoresis	Simulate gel electrophoresis for separation of DNA fragments
Ligation	Apply ligation algorithm to DNA from all selected nodes to generate ligation products
Screening	Use feature screening (simulates e.g. antibiotic resistance) or PCR screening to pick DNA of interest from a mixture
	Feature screening: MCDS detects all features in the substrates and list them in the property panel. Users can specify a combination of features and number of appearance. MCDS will work out the DNAs that match the conditions
	PCR screening: MCDS will use each of substrate to produce PCR products with given primers. Substrates that can generate PCR products within specific length range will be selected
Recombination	Apply recombination algorithm to DNA from all selected nodes to generate recombination products
Restriction enzyme analysis	Apply restriction enzyme analysis to DNA form all selected nodes. Users can set up conditions
Sequence merging	Use merging algorithm to merge two sequences with overlapping ends (e.g., overlapping sequencing results). Generates all possible outcomes if multiple homologous sequences are present
Sequence designer	Create a node that allows the user to design any DNA sequence by typing, pasting, or using a feature
	The designer code syntax can be found in the table online: https://goo.gl/qNdZa0. There is also an alternative UI sequence designer
Sequence information hash code selection	This function assigns a ‘hash code’ to all sequence products of a function (e.g. ligation, recombination). It allows the user select desired sequences where length or features (e.g. selectable markers) cannot be used to differentiate between target fragments
Sequencing simulation	Create a node that can generate the theoretical sequence of a one-primer sequencing reaction
Sequence comparison	Create a node that compares DNA from selected nodes. In the sequence comparison node, users can select one sequence as template and other sequences will be aligned to the selected sequence. Both features and comparison alignments are be shown in the sequence viewer and vector map to allow users to check if an alignment (i.e. sequencing result) covers a feature (i.e. gene)
Host	Create a node which define a host cell. Useful to confirm that specific hosts can e.g. host target plasmids, support conjugation, etc
Transformation	Transform DNA from select nodes into the selected (substrate) host cell.
incubation	Perform incubation procedure for select nodes. The ‘overnight incubation’ mode can simulate antibiotic screening
Extraction	Extract all plasmid DNA from selected nodes that are hosts (cells).
Gibson assembly designer	Design primers (based on user preferences) and apply Gibson assembly to DNA from selected nodes. Each source node must have only one DNA molecule. Where PCR products are used in a source node, the primer sequences will automatically be modified by extension to provide the correct overlap sequence
CRISPR digestion	Perform CRISPR digestion by using selected sgRNA and substrates

**Table 2:** Additional software features. These additional features are available from various locations, including drop-down menus, the Property panel and the Context menu.

**Additional feature**	**Details**
Supported file types	Genebank DNA sequence (.gb), Sequence text file (.txt), Sequence file (.seq), Sequencing result (.ab1,. scf). MCDS DNA file (.vct), MCDS project (.vxt)
Sequence import	Load sequence with the “Load Sequence” button
	Copy sequence files (.gb .txt .seq .ab1.scf) in Windows file explorer and paste into MCDS flowchart view
Restriction enzymes	Restriction enzymes can be managed and customized in “Restriction Enzymes” button in the “Settings” menu
Recombination sites	Recombination sites can be managed and customized in “Recombination Sites” button in the “Setting” menu
gRNA Sites	gRNA sites can be managed and customized in “gRNA Sites” button in the “Setting” menu.
Codon table	MCDS can download all genes of an organism from KEGG and calculate codon usage.
	Selected codon table is used in “Translation” and “Codon optimization”
Digestion buffers	MCDS manages digestion buffers by supplier and product name so as to describe the difference between buffers and commercial names (e.g. buffer specificities of BcuI from Thermofisher and SpeI/SpeI-HF from NEB can be all stored in MCDS)
	MCDS stores the buffer activity table and can automatically calculate the optimal buffer for a combination of enzymes
Feature setting	All features found in genebank files, vector (vct) files and pasted files (when copied from one node to another, or Windows Explorer) will be automatically included in the project.
	In all DNA generated in MCDS, features will be automatically annotated
	Features can be managed and edited in “Manage Features” of the dashboard
Enzyme setting	User can type or select enzymes in the dashboard, and all enzyme sites will be shown in all the vector maps
Node status	Users can specify the status of a node: “Not Started”, “In Progress”, “Finished” or “Obsolete”. Node background will be changed to a corresponding color to present the status of node in the flowchart
Sequencing data	Users can embed sequence result files into the “Sequencing Results” of a node and data traces can be displayed. Embedded files can be exported
Figure file	Users can embed images such as gel figures into the “Gel Figures” tab. Embedded files can be exported
Primer management	MCDS can maintain a list of primers. In the project summary, MCDS detects recorded primers so that users can only send newly designed primers into clipboard
Primer designer	Primer Search: users can first select a region in the sequence viewer or vector map and then click “Find” to search for a pair of primers with Tm above given value and with minimal hairpins, dimers, cross dimers to amplify the selected region. Additional candidates are listed in the drop button of the “Find” button
	One click primer design: users can click the row above or below the double-strand sequence in the sequence viewer to design a primer with 5′ end starting at the clicked nucleotide and with Tm above give value
	Gibbs free energy change values and affected nucleotides are shown for hairpins, dimers, cross dimers and priming sites
Ligation and recombination algorithms	Ligation and recombination are the same algorithm with different “Connection” and “Cyclization” methods
	Supported recombination: Lambda red recombination, homologous, Gibson assembly
	Normal mode: users can specify how many rounds the algorithm should be applied to the substrates
	Exhaustive mode: users can specify how many copies of each fragment should be used in the assembly. The algorithm will try to find all possible products that use up all given fragments
Online database	This function can be accessed from the button or main menu. Users can search and download DNA sequence from KEGG and NCBI in MCDS. Obtained sequences will be added to the flowchart as nodes
Node status tracking	Users can specify the status of a node as “Not Started”, “In Progress”, “Finished” or “Obsolete”. The status of a node is shown by its background color (white, red, green or gray)
Group copy and group paste	Group Copy and Group Paste can be accessed from “Copy Group” and “Paste Group” buttons or context menu. Group Copy copies all selected nodes with their relations in the flowchart. Group Paste pastes the copied nodes and relation into a flowchart and automatically load the features in nodes if the features are not yet included in the project
ORF detection	ORF detection can be accessed using the Context menu by right-clicking when one or more nodes are selected. It detects all ORFs that meets the specified conditions and will draw them in the node. Features that are not recorded in the feature setting (including any unsaved ORFs in the previous detection) will be removed during the ORF detection.
	In order to save the detected ORFs to the feature setting, the detected ORFs of interest must be managed in a sequence viewer of the Property panel.

### 3.1 Interactive flowchart for workflow management

To enable an overview with simple access to any step of a multiple-step workflow simulation, an interactive flowchart view was implemented. This flowchart is the main user interface (UI) view of an MCDS project file. In this flowchart view, each operation is presented as a node with arrows intuitively illustrating the relationships between each node. By selecting a node in the flowchart, the corresponding details of the node are presented in the ʻProperty Panel’. Convenient CAD features (including add, remove, drag-and-drop, multiple select, copy and paste of nodes, view scrolling, dragging and smooth mouse-wheel zooming of views) are all implemented. In particular, features such as automatic layout, and dragging a node together with its downstream branch are also supported to take the advantage of flowchart style design. In a given node, the node ID, name, operation method, a DNA vector map (if a node contains only one DNA molecule), the summary of results, and comments are shown in rectangular box, providing an overview of operations and results. In addition, for nodes showing restriction enzyme digestion, enzymes and compatible buffers are also listed to guide restriction enzyme digestion.

### 3.2 Printing and exporting flowchart data

The PrintPage function allows for printing of any part of the flowchart. In the ʻprint’ mode, a ʻPrint Page’ box can be added and dragged to anywhere of the flowchart, and then zoomed to the appropriate size to print the indicated part of the flowchart. In addition to printing, either the whole or any selected part of the flowchart can also be exported as EMF vector drawing or copy-and-pasted into document editors (e.g. Word, Power Point or Visio).

### 3.3 Flowchart nodes

A flowchart node represents a step of operation. The operations include DNA molecule, PCR, gel electrophoresis, solution extraction, enzyme modifications, ligation, PCR and feature screening, sequence comparison, sequencing simulation, merging of sequence results, recombination (including site-specific recombination, homologous recombination and recently developed in vitro recombination such as SLIC, In-Fusion, Gibson Isothermal Assembly, yeast recombination assembly, recET homologous recombination), restriction site analysis, DNA sequence design, transformation, host selection, incubation, DNA extraction. This functionality can be extended to support more operations for future technologies. The parameters of a flowchart node are maintained in a ʻDNAInfo’ class, which contains all necessary information of an operation.

### 3.4 The ʻProperty’ panel

The Property panel, located at the bottom of the project tab, is the user interface for setting up the parameters for a selected node. It allows the users to edit the node ID and name, choose the precursors for a node, modify the status, apply or abandon changes, and browse the summary of resulting DNA molecules. For each specific operation, there is a tab panel to edit available parameters. A single-click on the ʻApply’ button starts the simulation of the node operation.

### 3.5 Vector view, sequence view and in situ primer designer

By clicking the ʻView DNA’ button of the ʻProperty Panel’, details of all product DNA sequences are presented as tabs in the property panel. Features such as restriction enzyme sites and other annotated regions are plotted in both sequence and vector views. The user can select the feature or any part of the sequence in either the vector map view or the sequence view.

MCDS can automatically calculate primer melting temperatures (T_*m*_) to design minimal primers. By clicking the ʻPR’ button in PCR design and PCR screening nodes, users can select primer sequences with no less than the given T_*m*_ value directly ʻin situ’ in the sequence viewer. MCDS will identify and list primers that can amplify the selected region of DNA when a region of DNA sequence is selected and the ‘Find’ button is clicked. The primer list can be accessed using the dropdown arrow in the ‘Find’ button.

### 3.6 Ligation algorithm

**Fig. 3:**
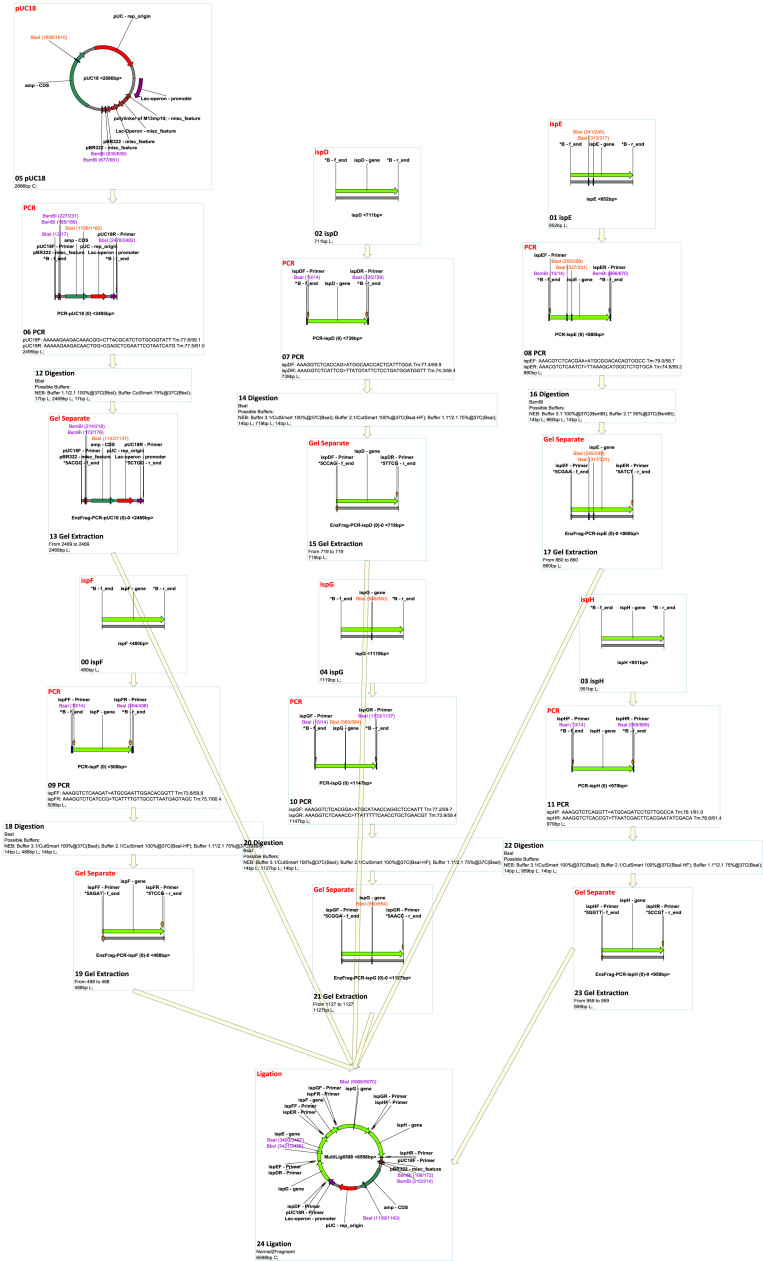
MCDS design and simulation of Golden Gate assembly.

Ligation is the basic operation in recombinant DNA technology (Lobban and Kaiser, 1973), and is supported by the ‘Ligation’ node type in the flowchart. In the ligation algorithm, two ends can only be ligated when they meet the following two conditions: 1) recognition of matching ends; at least one of the two ends should contain a phosphate; 2) either both ends are blunt, or both overhangs are 3′ or 5′ and are complementary. The ‘normal’ mode simulates real ligations with specified complexity, where users can specify how many times that fragment can be used in the ligation. The ‘exhaustive’ mode simulates ligation-based multiple DNA assembly technologies such Golden Gate Assembly (Engler et al., 2009, Engler and Marillonnet, 2014, Marillonnet and Werner, 2015), where only products that contain a specified number of all fragments are generated. Fig. 3 and Supplementary file 2 show an example of Golden Gate design and assembly.

### 3.7 Recombination algorithm

To simulate recombination properly, the molecular mechanisms of different types of recombinations were studied. This revealed that a shared ‘core sequence’ was always found in specific types of recombination, as follows: 1) homologous recombination (Aylon and Kupiec, 2004, Takahashi and Norden, 1994), Lambda Red recombination (Maresca et al., 2010, Poteete, 2008), Gibson Assembly (Gibson, 2011, Gibson et al., 2009), SLIC (Li and Elledge, 2007), yeast homologous assembly (Noskov et al., 2003) and redET homologous recombination (Fu et al., 2012) always require a shared (homologous/core) fragment for two substrates to recombine; 2) site-specific recombination sites also have a shared core sequence. Although the Holliday junction intermediates of tyrosine family site-specific recombinases such as lambda attachment sequence recombination (Cassell and Segall, 2003, Radman-Livaja et al., 2006), cre-loxP (Van Duyne, 2001) and FLP-frt (Chen et al., 2000, Pan et al., 1993) system do not generate real single-strand overhangs, the cut and reconnection at their core sequences are very similar to the digestion and ligation of double-strand DNA. The serine recombinase family of recombinase systems (such as ΦC31) are slightly different in mechanism; by forming a protein-DNA bond (Reed and Grindley, 1981, Thorpe et al., 2000), cleavage and reconnection of the core sequence are also engaged.

To support these observations, recombination are simulated using a virtual digestion and ligation method, as follows:

1) Virtual digestion. Recombination sites are treated as virtual restriction digestion enzyme sites and the digested to generate virtual overhangs at the shared sequence; the virtual digestion overhangs of reversible recombination sites are labeled with the same markers so they can be joined back together, while the virtual digestion overhangs of irreversible recombination sites are labeled with different markers to stop them from being assembled back to original substrates. In the case of site-specific recombination, the recombination sites are treated as virtual restriction enzyme sites and the shared core sequences are digested into virtual single-strand overhangs; in homologous recombination, the homologous sequences shared by substrates are first identified, then each homologous sequence is treated as a virtual restriction site. For lambda Red recombination, homologous sequences within 20–100 bp at both ends of a linear DNA are identified first and treated as virtual restriction sites; for SLIC, Gibson Assembly and yeast recombination, homologous fragments within 250 bp from the end of DNA are indentified and treated as virtual restriction sites. For all types of homologous recombination, the whole homologous sequences are digested into virtual overhangs.
2) Virtual ligation. Similar to the ligation algorithm, for both site-specific recombination and homologous recombination, there are also ʻnormal’ and ʻexhaustive’ modes. However, in both modes all of the intermediates with virtual overhangs will be disposed of at the end of calculation to ensure only real DNA products are shown in the final products.

**Fig. 4:**
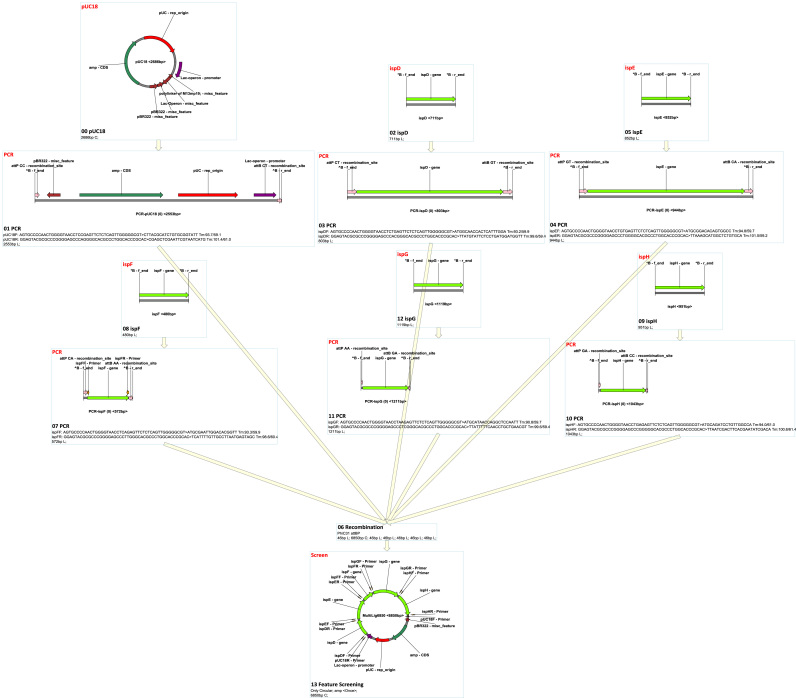
MCDS design and simulation of ΦC31 Assembly.

**Fig. 5:**
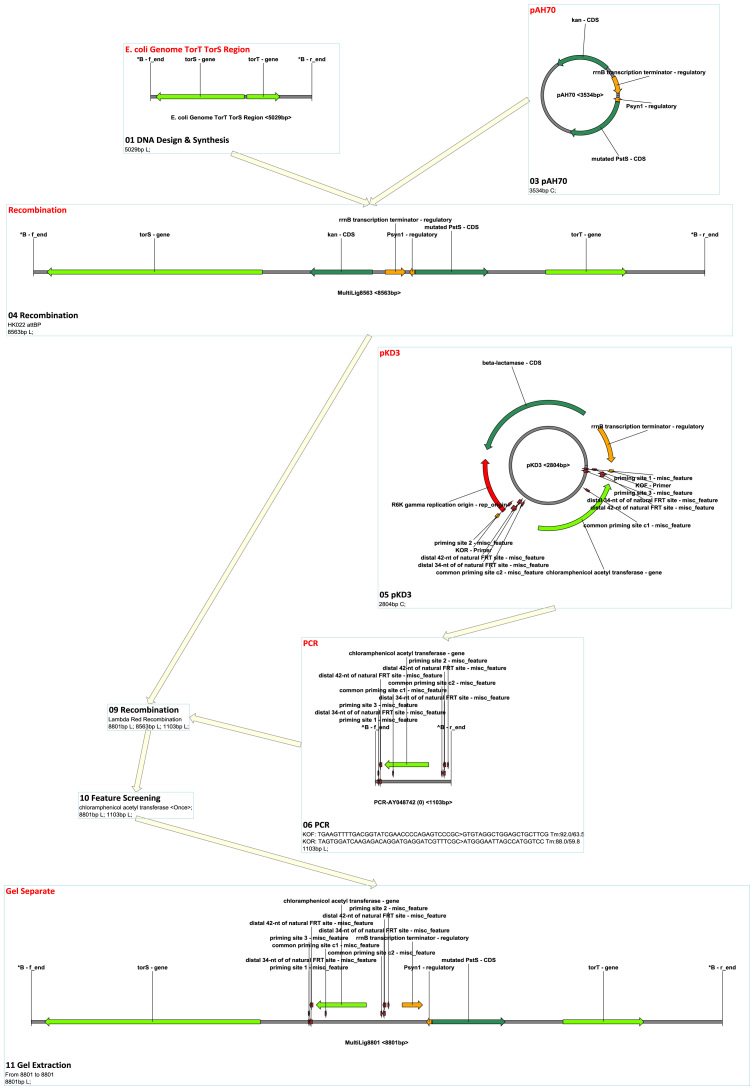
MCDS design and simulation of site-specific recombination and Lambda Red recombination.

There are four types of built-in homologous recombination supported by MCDS: ʻHomologous Recombination’, which simulates bacterial recA-dependent homologous recombination (Aylon and Kupiec, 2004, Takahashi and Norden, 1994); ʻLambda Red Recombination’, which simulates Lambda Red double-exchange recombination (Datsenko and Wanner, 2000, Sabri et al., 2013, Stringer et al., 2012, Wang et al., 2014); ʻIn Vitro Annealing’, which simulates assembly that does not cut off non-matching tails, such as SLIC (Li and Elledge, 2007); and ʻIn Vivo Annealing’, which simulates Gibson Assembly (Gibson, 2011, Gibson et al., 2009) and yeast homologous assembly (Noskov et al., 2003). Any site-specific recombination can be supported by adding corresponding recombination site definitions in the recombination site management (the management interface can be found in the ʻSetting’ menu). ΦC31 (Colloms et al., 2014) and PhiBT1 (Zhang et al., 2010) assembly can be simulated by adding their site definitions. Fig. 4 and Supplementary file 3 show an example of a ΦC31 assembly. Fig. 5 and Supplementary file 4 show an example of an HK022 integration and Lambda Red knock-out.

### 3.8 Gibson assembly designer

**Fig. 6:**
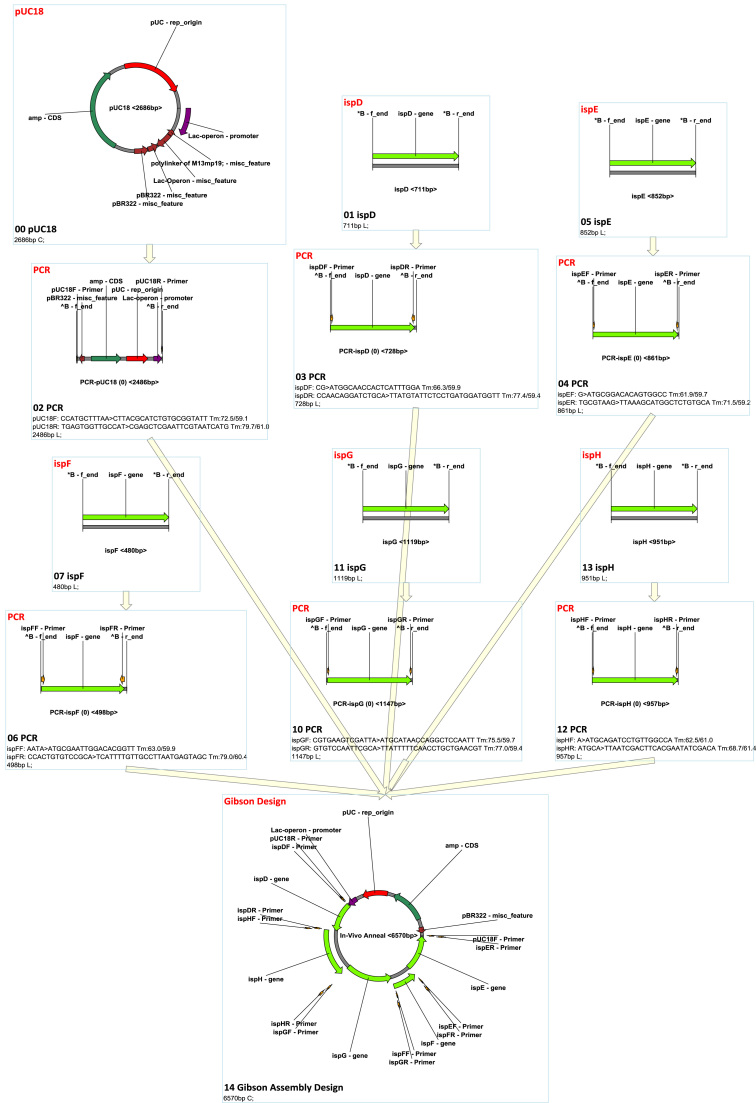
MCDS design and simulation of multiple fragment gibson assembly.

In the ʻGibson Assembly’ design node option, a source fragment will be treated differently if it is the product of a PCR node since PCR primers can be modified by the Gibson assembly design algorithm if they are not specified as ʻfixed’. The direction of assembly can be also specified. The Gibson Assembly design algorithm automatically searches for proper linkers and attaches them to the primers of PCR nodes that are not specified as ʻfixed’; it also and guarantees the melting temperature and length are above set thresholds. The Gibson Assembly product will be calculated using the ʻexhaustive’ mode of' in vivo recombination’. Fig. 6 and Supplementary file 5 show an example of a Gibson Assembly design and simulation.

### 3.9 CRISPR digestion

In the ʻCRISPR digestions’ design node option, the algorithm uses guide RNA (gRNA) to search targeting sequences in all source nodes and the DNA sequence containing the gRNA as the gRNA expressing vector, and then applies the targeting sequence with a Protospacer Adjacent Motif (PAM) to create a temporary'restriction enzyme’ so as to use the restriction digestion algorithm to digest the substrate DNA. Customized gRNA and PAM sequences can be defined in the gRNA manager to support more CRISPR enzymes, such as the recently discovered Cpf1 (Zetsche et al., 2015).

### 3.10 Feature screening

In a real cloning and recombineering experiment, positive clones are usually screened with selective markers such as antibiotic resistant genes or complementation of auxotrophic mutants. In order to simulate selective marker screening as per experimental procedures, a feature screening algorithm is implemented in the ʻScreening’ design node. This allows the user to select a constructed DNA from the mixture of ligation or recombination products by criteria such as whether the DNA is circular and whether the DNA contains a specific number of features.

### 3.11 PCR screening algorithm

In addition to (and sometimes instead of) selective marker screening, colonies are very commonly screened by PCR. A PCR screening algorithm is included in the ʻScreening’ design node to facilitate simulation of this. In PCR screening mode, the user can design a pair of primers with the sequence viewer and primer designer described above (Section 3.3) and specify the length range for expected products. The PCR screening algorithm applies the primer set to each of the source DNA molecules and selects the DNA molecules that can produce a PCR product within the specified product length range.

### 3.12 Sequence comparison algorithm

Sequence comparison is essential for the analysis of clones and constructed vectors. It is usually performed using Basic Local Alignment Search Tool (BLAST) (Altschul et al., 1990). However, BLAST outputs do not usually show DNA sequence features, making it a time-consuming process to identify mutations in sequences with multiple complex features. In order to provide immediate access to features in alignment files, a ʻSequence Comparison’ was developed with the algorithm to present fragments of alignments (including matches, insertions and deletions) as features in the DNA sequence view and the DNA vector map. Sequencing results can be loaded into the interactive flowchart by copying ʻSCF’ or'AB1′ files from Windows File Explorer. By selecting both the sequencing result node(s) and the theoretical (simulation) sequence node (usually a node presenting a constructed plasmid) and creating a ʻSequence Comparison’ node, multiple sequencing results can be presented on the same theoretical sequence vector map. This allows the user to immediately identify problematic mutations and select successful clones.

### 3.13 Free DNA design

A dramatic reduction in cost over the last decade has significantly increased the use of DNA synthesis technology, making it routine in many laboratories. A code-based ʻDNA sequence designer’ node is included to support the direct input of DNA sequences, the use of defined features in the feature management, and the usage of defined restriction enzyme sites (see Supplementary table 1). This unique system allows DNA features (including overhangs and hairpins) to be annotated and maintained with the sequence throughout the design process.

### 3.14 Visualized sequence and primer designer

In order to keep the logical design of both sequences and primers, a visualized sequence and primer designer was implemented in the ʻsequence design’ and'PCR’ nodes. In this visualized designer, protection sequences, restriction sites, amino acid residues, DNA features, and matching sequences can be recorded as visible ʻblocks’ and configured by connecting their ends. In addition, one-amino acid-one-codon algorithm and guided-random algorithm were both implemented for sequence design from an amino acid sequence (Puigbo et al., 2007).

### 3.15 Workflow design automation

Existing cloning software packages cannot be used to quickly and easily implement changes in the design workflow and allow propagation of those changes throughout the downstream process. To allow this functionality in MCDS, an algorithm to recalculate nodes along the flowchart topology structure was implemented. By double-clicking the “Apply” command or clicking the ʻRecalculated all children’ button in the context menu, all downstream nodes of the selected node will be recalculated in order.

### 3.16 Restriction enzymes management, feature management, host management and primer management

Tabs can be set up in the ʻProperty’ panel to display different types of information sets, including all that are used in all DNA maps, all the features, all the cloning hosts, or all of the primers. The selection of restriction enzymes and features in these tabs will affect the display of all restriction enzymes and features shown on the each DNA map of the project. For any newly-generated DNA maps (including uploaded sequencing results), the selected restriction enzymes in ʻenzyme management’ and defined features in ʻfeature management’ will be automatically annotated. This makes it easier for user to determine whether the products are correct according to desired features and restriction enzymes.

### 3.17 Notes and node status

In order to enable the management of a complex project, a workflow management feature was implemented to display user notes and status color of a workflow node. Notes can be added to a text box in the bottom left of the property panel of a node. Four node statuses -‘not started’, ‘in progress’, ‘completed’ and ‘obsolete’ - can be set at the top right of the property panel. Each status corresponds to a background color and enables users to see the status of the node directly from the workflow overview of the project.

### 3.18 Project summary and primer management

In order to provide a classified overview of a whole project, a summary function was implemented in the dashboard of a project view, accessible by clicking the ‘View Summary’ button. In the ‘summary tab’ panel, primers, double-strand DNA to synthesize, restriction enzymes and modification enzyme are all listed. MCDS keeps a record of all primers ordered so as to check whether a primer has already been ordered. The ‘Copy New’ button allows the user to copy only the newly designed primer name and sequence to the Windows clipboard. This functionality enables users to focus only on the design and access the new primers afterwards using just a few clicks.

### 3.19 Description of various types of DNA molecule

DNA molecules engaged in cloning and recombineering are not just linear or circular, but can be also a fragment of genome or a hairpin-end linear DNA (Cui et al., 2007, Ravin, 2011). In order to support all type of DNA molecules that can be encountered, a set of codes for presenting DNA molecule ends was implemented. Details for this code are shown in Supplementary table 2.

### 3.20 Restriction enzyme digestion and digestion buffer search algorithm

In order to support different nomenclature and various digestion buffer systems from different companies (including the ‘normal’ and HF versions of NEB restriction enzymes), a DNA digestion buffer search algorithm that uses an alias name system for restriction enzymes was implemented. In the ‘digestion buffer’ setting, a digestion buffer system of a given company retains the company product name and digestion efficiency in each buffer from the buffer system. For example, SpeI from Thermofisher is recorded under the alias name ‘BcuI’, which maps to SpeI; and SpeI and SpeI-HF from NEB are recorded under two different alias name entries, both of which map to SpeI. Consequently, all entries mapped to SpeI will be used in the digestion buffer searching algorithm when SpeI is engaged in a digestion node.

### 3.21 Enzyme analysis algorithm

In order to search for restriction enzymes that can meet multiple complex conditions, the ‘enzyme analysis’ algorithm was developed for the ‘restriction enzyme analysis’ node. This algorithm allows users to select whole circular plasmid or any part thereof as source DNA molecules and define the conditions of appearance of a given enzyme site (greater than, less than or equal to a specified number). The algorithm will test all those conditions on each of the defined restriction enzymes and generate a list of enzymes that match all of those conditions.

### 3.22 PCR algorithm

PCR is the most widely used molecular biology technique (Saiki et al., 1985). To simulate a real PCR process and generate overlap extension products and by-products (Ho et al., 1989, Horton et al., 1989) rather than copying a region of DNA sequence between two binding sites, the PCR algorithm in MCDS calculates all possible single-strand products that can be produced from substrates. The final PCR products are calculated by annealing single-stranded DNAs with matching 3′ regions. In the ‘visualized primer designer’ mode, PCR reactions with one single primer or more than two primers can also be simulated.

### 3.23 Name-to-sequence replacement in primer editing boxes

On pressing ‘Enter’ key, the primer editing boxes in Property Panel for PCR or PCR screening nodes will replace restriction enzyme and recombination site names in ‘[*name*]’, ‘[*name*:*pattern*>‘ or ‘<*name*:*pattern*]’ (e.g. [BamHI],<phiC31 attB:GC]) to their corresponding sequences while replacing the ‘N’ with provided pattern after ‘:’. For palindromic sequences, ‘[*name*]’ should be used; for non-palindromic sequences, ‘[*name*:*pattern*> ‘should be used for forward sequence and ‘<*name*:*pattern*]’ should be used for reverse-complemented sequence.

### 3.24 Gel separation algorithm

To simulate gel separation and DNA selection by length, a gel separation algorithm was implemented in the ‘gel separation’ node. This allows users to specify a length range and will select source DNA molecules within that range. Fragments that are shorter than 50 bp or 50 nt are ignored.

### 3.25 T*_m_* Viewer

The T_*m*_ Viewer allows visualization of the melting temperature of each section of a DNA fragment, thus allowing users to identify potential problems in PCR amplification. The T_*m*_ Viewer can be accessed using the Context menu by right-clicking a node. It calculates the T_*m*_ value for every oligonucleotide fragment of specified length in the DNA molecule and displays the T_*m*_ in a column view.

### 3.26 Bacterial host and incubation simulation algorithm

To simulate plasmid replication conditions and real antibiotic selection/incubation procedures, support for bacterial host features, including primases for replication origin and antibiotic resistance genes, are implemented as text commands. The ‘Incubation’ algorithm searches replication origins that match the primase commands and the antibiotic resistance features that match the antibiotics added to the incubation culture.

In addition, in the ‘bacterial host’ node, genomic fragments can be set up. The genomic fragments can be used as substrates for in vivo digestion and targets for recombination. This allows the simulation of complex in vivo recombineering.

### 3.27 Transformation

To simulate common transformation techniques, a transformation algorithm supporting chemical transformation, electroporation, and conjugative transformation was implemented. To further generate cells with different types of combinations when multiple plasmids were transformed, three different transformation options – ‘All into one Cell’, ‘Each DNA per Cell’ and ‘Combinational’ – are supported by the transformation algorithm. For ‘All into one Cell’, all the DNAs will be transformed into only one host cell. This approach fits most of the simple construction scenarios. For ‘Each DNA per Cell’, each DNA will be transformed to a single cell. This option works for construction of libraries. The ‘Combinational’ algorithm will iterate all possible combinations of DNA and cells.

### 3.28 Plasmid extraction

To simulate plasmid extraction, a plasmid extraction algorithm was implemented in the ‘plasmid extraction’ node. All DNA that are hosted in a cell (excluding the host genomic DNA) can be extracted as mixture of DNA molecules.

### 3.29 Support for file types

MCDS supports use of multiple file types. Genebank files, sequencing result files such (either AB1 or SCF), DNA sequence files (.seq) can all be copied from Windows File Explorer and pasted into a MCDS project workflow view. MCDS can also present the sequencing data traces from sequencing result files (AB1 and SCF).

### 3.30 Open Reading Frame detection

The ORF (Open Reading Frame) detection algorithm allows the detection of ORF in DNA sequences with specified start codons and minimal ORF length. It can be accessed using the Context menu by right-clicking a node. Detected ORFs will be drawn in the node, and they can be added to the feature setting by first selecting the ORF in any sequence viewer of the Property panel and then managing the ORF via the ‘Manage Feature’ button in menu strip on the top right of the main window.

### 3.31 Automatic update

In order to keep MCDS updated to the latest version, an automatic search for new versions occurs every time MCDS is started. If a new version is found, MCDS will shut down and run ‘MCDS Update. exe’ to download the new version of MCDS.

### 3.32 Application and practice in molecular cloning

MCDS was used previously to successfully design, simulate and guide a complete project. The files can be download from Supplementary Files S1-S12 links from the webpage http://doi. org/10.1371/journal. pone.0056854 (Shi et al., 2013). These files can be used as a practice example.

## 4 Discussion

### 4.1 Interactive flowchart user interface for design, simulation, reuse, tracking and management of complex workflows

Current cloning simulation software packages use individual files for each plasmid/constructed sequence, requiring the user to maintain project design and simulation information manually. To achieve project overview and workflow simulation, MCDS compiles all related sequence information into one file per project and has an interactive visual flowchart GUI. The flowchart can be freely dragged, aligned, scrolled, zoomed, selected and moved to allow users an overview of the whole project or workflow, or to check/design each individual step (node). Below the flow chart is a ‘Property’ panel, which is editable and contains all necessary detailed information for the selected node. Each node is connected to node(s) that provide substrate(s) for its operation by arrows.

MCDS achieves the simulation of workflow by automatic recalculation of nodes in workflow using one command. By clicking the ‘Recalculate All Children’ in the Context menu (available by right-clicking anywhere in the flow chart) or double-clicking ‘Accept’ in the Property panel, all nodes downstream of the selected node in the workflow can be automatically recalculated based on the new result of that node. Users can duplicate individual nodes or a workflow of nodes by using the ‘Group Copy’ and ‘Group Paste’ buttons so as to reuse/redesign a workflow.

Through the features described above, the interactive flowchart addresses the project overview and workflow limitations of previous ‘file list-based’ software programs. Overall project management is much more user-friendly, and the probability of mistakes during design or experimentation due to lost/forgotten information is decreased.

### 4.2 Simulation of cloning and customized recombineering technology

There are 20 types of operations (functionally displayed as nodes) available for application in the interactive flowchart (Table 1). Digestion, ligation and recombination algorithms of MCDS are novel and unique, and are based on molecular mechanisms of these processes so as to truly simulate cloning and recombineering. This means that instead of producing only one product with minimal simulation, the ligation and recombination algorithms in MCDS will generate a range of possible intermediates and by-products (in ‘normal’ mode) or all possible products (in ‘exhaustive’ mode), and then use feature information or PCR selection (as directed by the user) to screen the product of interest from other intermediates and by-products. These screens simulate marker gene selection or colony PCR.

By editing the settings of restriction sites, recombination sites and gRNA sites, users can add customized support for additional site-specific recombination approaches that are not commonly used (such as phi80 and P22 attachment sequences recombination (Leong et al., 1985), and R4 (Matsuura et al., 1996) and ϕBT1 (Gregory et al., 2003) integrases). The available ligation/recombination algorithms and customizable sites provide support to most of the newer recombineering technologies, including lambda Red recombination (Datsenko and Wanner, 2000, Sabri et al., 2013, Stringer et al., 2012), landing-pad integration (Kuhlman and Cox, 2010), site-specific recombination(Haldimann and Wanner, 2001, Shi et al., 2013), ΦC31 assembly (Colloms et al., 2014), CLIVA assembly (Zou et al., 2013), and in vivo CRISPR digestion (Jiang et al., 2013).

## 5 Conclusion

MCDS is an all-in-one in silico design, simulation and management platform that has addressed the overview, workflow, support for new technologies and management limitations we identified in currently available software programs. The software was used previously to successfully design, simulate and guide a complete project (Supplementary files S1–S12 from http://doi. org/10.1371/journal. pone.0056854) (Shi et al., 2013). The architecture of the flowchart node GUI and algorithm separation enables incorporation of any new technology by simply adding support for new type of nodes and algorithms. Through this, MCDS aims to support all new cloning technologies available now or in the future, and comes with a built-in automatic update function to make updates available to the community.

## Footnotes

Appendix A Supplementary data associated with this article can be found in the online version at doi:10.1016/j. meteno.2016.05.003.
